# Evolution of the statistical distribution in a topological defect network

**DOI:** 10.1038/srep17057

**Published:** 2015-11-20

**Authors:** Fei Xue, Xueyun Wang, Ion socolenco, Yijia Gu, Long-Qing Chen, Sang-Wook Cheong

**Affiliations:** 1 Department of Materials Science and Engineering, The Pennsylvania State University, University Park, Pennsylvania 16802, USA; 2 Rutgers Center for Emergent Materials and Department of Physics and Astronomy, Rutgers University, Piscataway, New Jersey 08854, USA

## Abstract

The complex networks of numerous topological defects in hexagonal manganites are highly relevant to vastly different phenomena from the birth of our cosmos to superfluidity transition. The topological defects in hexagonal manganites form two types of domain networks: type-I without and type-II with electric self-poling. A combined phase-field simulations and experimental study shows that the frequencies of domains with N-sides, i.e. of N-gons, in a type-I network are fitted by a lognormal distribution, whereas those in type-II display a scale-free power-law distribution with exponent ∼2. A preferential attachment process that N-gons with a larger N have higher probability of coalescence is responsible for the emergence of the scale-free networks. Since the domain networks can be observed, analyzed, and manipulated at room temperature, hexagonal manganites provide a unique opportunity to explore how the statistical distribution of a topological defect network evolves with an external electric field.

A network (graph) is a representation of a set of objects (nodes) with connections between them^1^. A complex network is a network with non-trivial topological features, which are absent in simple networks such as regular lattices and classical random graphs^2^,^3^,^4^. The great majority of real-world networks, including World Wide Web, the Internet, movie actor collaboration networks, neural networks and many others, are complex networks^3^,^5^,^6^. Some complex networks demonstrate a scale-free power-law distribution of connections (degrees), which attract enormous attention due to the notable characteristics such as relative commonness of nodes with a degree that significantly exceeds the average and a small average distance (a small number of hops) between two nodes^6^,^7^.

Multiferroic hexagonal REMnO_3_ (RE, rare earths) exhibits fascinating topological defects produced from a structural phase transition well above room temperature^8^,^9^,^10^,^11^,^12^,^13^. The transition is manifested by a structural trimerization giving rise to three types of antiphase domains (α, β, γ) with each exhibiting two possible directions of induced ferroelectric polarization (+, –) along the c axis^14^,^15^. Therefore, there are a total of six types of antiphase and ferroelectric domains, the co-existence of which leads to the formation of topological defects, i.e. vortex lines in three dimensions (3D) or vortices/anti-vortices along the basal plane (2D)^12^,^16^,^17^. The vortices, anti-vortices, and domain walls form a complex network in 2D, which can be analyzed using the graph theory, a mathematical tool for analyzing the nature of connectivity^1^,^17^. In the graph theory, the vortex and anti-vortex cores are described as nodes, the domain walls as edges, and the domains as faces/regions. In the intriguing domain networks of REMnO_3_, all the nodes have six edges connected to them, and a domain is surrounded by N (an even integer) nodes/edges and thus called an N-gon. The domain network of vortices can be categorized into two types: type-I networks with statistically equal fractions of the six types of domains, and type-II networks with a preferred electric polarization direction either along the positive (+*c*) or negative (–*c*)^17^. Type-II networks result from poling by external electric fields or self-poling induced by chemical gradients, e.g. the concentration gradients of chemical defects^18^.

Numerical simulations based on phase-field methods can not only predict the domain patterns and topological distributions, but also their temporal evolutions as well as the detailed topological changes^19^,^20^. The simulation results allow us to efficiently perform statistical analysis on large datasets. Here both the simulations and experiments show that type-I networks are fitted by a lognormal N-gon distribution with the logarithms of its numbers normally distributed, in contrast to the scale-free power-law distribution in type-II networks. Detailed analysis based on the simulation results demonstrates that a preferential attachment process, i.e. a process that the N-gons with a larger N have higher probability to coalesce with other N-gons during transition from type-I to type-II networks, is responsible for the appearance of the power-law behavior.

First, the phase-field method^20^ is employed to simulate the temporal evolution of spatial domain patterns. The trimerization of the hexagonal REMnO_3_ is caused by the displacements of related oxygen atoms, which can be described by the magnitude *Q* and the azimuthal angle Φ^21^. In the phase-field simulations, the polar coordinates *Q* and Φ are transformed into Cartesian coordinates 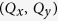, where 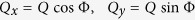. The trimerization induces a polarization *P*_*z*_, a secondary order parameter. Based on the hexagonal symmetry, the total free energy density is given by^21^,^22^,^23^


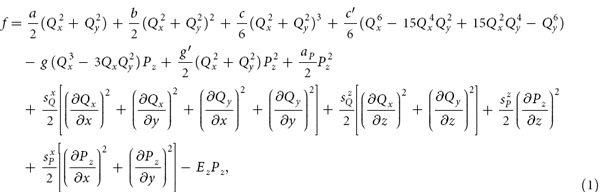


where 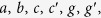
and *a*_*p*_ are the coefficients for the Landau free energy function, 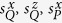, and 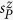 are coefficients for the gradient energy terms, and *E*_*z*_ is an external electric field along the z direction. We employ YMnO_3_ as an example with all the parameters from first-principles calculations^21^. The phase-field equations are solved with the initial condition of zero plus a small random noise for the order parameter components^24^. Periodic boundary conditions are applied along the three directions. The system size is 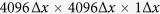
(unless otherwise noted), and the grid spacing is 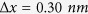.

In the absence of an external electric field, the phase-field simulation generates a type-I network with six domains around a vortex or anti-vortex, as shown in Fig. 1. Isolated domains exist as inclusions embedded in another domain, e.g. 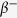 within 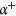
domains^17^. The presence of the isolated domains reflects the six degenerate energy minima in the free energy landscape, in contrast to the continuous symmetry in the X-Y model^25^. From the phase-field simulations, the isolated domains are created during vortex-antivortex annihilations as shown in Fig. 2(a–c) and as demonstrated by Movie-I (system size 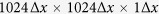) in the supplementary materials.

Another consequence of the six-fold energy degeneracy is the connected domain networks and the statistical distribution of the N-gons. Experimentally we perform N-gon analysis on three REMnO_3_ samples, all grown using the standard flux method with the details given in earlier reports^17^,^26^. Large-region optical images are taken after chemical etching (for details, see supplementary materials), and the results from N-gon analysis are summarized in Fig. 3(a–c). As shown in Fig. 3(a), the type-I networks can be approximated by a lognormal distribution 
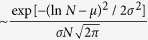
, where *μ* and *σ* are the mean and standard deviation of the corresponding normal distribution (for details of statistical analysis, see supplementary information). According to the graph theory^1^, the dual graph of a graph is a graph that has a node corresponding to a face of the original graph, and an edge joining two neighboring faces of the original graph. As shown in Fig. 2(d), in the dual graph of a type-I network, all the faces are 6-gons (see supplementary Fig. S6 for a dual graph of large-range domain patterns). When a node has N connections to other nodes, it is also called that the node has a degree of N^1^. The lognormal distribution of the N-gons in type-I networks indicates that the corresponding dual graph shows a lognormal degree distribution, which is also observed in other networks such as protein interaction networks^27^.

On the other hand, a type-II network is better approximated by a power-law distribution (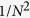), as shown in Fig. 3(c). Note that the average side of the N-gons following a power-law 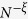 distribution is given by 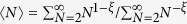, which is convergent with 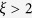 and divergent with 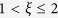, since 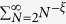 is convergent with 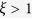 and divergent with 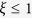. Therefore, 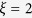 is the critical exponent below which 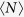 is divergent. The underlying mechanism of the critical behavior needs further investigation.

Since there exists a self-poling effect near the surfaces of REMnO_3_ crystals due to the concentration gradient of oxygen content, the surfaces often show type-II networks with type-I networks inside^17^,^28^. The surfaces of REMnO_3_ crystals annealed to ensure uniform oxygen content throughout the crystals tend to exhibit type-I patterns. However, we have infrequently observed an intermediate state between type-I and type-II networks, as shown in Fig. 3(b).

To reveal the underlying mechanism for the different statistical distributions in the type-I and type-II networks, we performed phase-field simulations of domain pattern formation and the N-gon analysis of predicted domain patterns generated with or without an applied electric field of 1200 kV·cm^−1^ (the magnitude of the electric field in the simulation is larger than the experimental saturation field of ~400 kV·cm^−1^ in P-E loops^29^). During the domain evolution under an applied electric field, it is assumed that the vortex cores are pinned by defects and have zero mobility. It should be noted that the mobility of the vortex and anti-vortex cores are temperature-dependent, although all the coefficients in equation (1) are assumed to be independent of temperature. At high temperatures, the vortex and anti-vortex cores are expected to be mobile, which is the case of Fig. 2(a–c). However, at room temperature, according to experimental observations, the mobility of the vortex and anti-vortex cores is very low^28^,^29^.

The results from the N-gon analysis of domain structures predicted by phase-field simulation are shown in Fig. 3(d–f). The dependence of the frequencies of the N-gons versus N agrees well with that obtained from experiments shown in Fig. 3(a–c). A type-I network from the phase-field simulation without electric fields exhibits a lognormal distribution, as shown in Fig. 3(d). Under the electric field, the slope of the curve corresponding to the electric-field-favored domains becomes smaller, whereas that corresponding to the electric-field-unfavored domains becomes larger, as shown in Fig. 3(e). Finally a type-II network is obtained, and the N-gon distribution of the three types of domains favored by the electric field exhibits a power-law distribution as shown in Fig. 3(f). The phase-field simulations on YMnO_3_ show the same statistical behaviors with the three REMnO_3_ samples with different elements in the RE sites and different system sizes. This indicates that the statistical behavior of the connected networks is intrinsic and universal for all REMnO_3_ systems and is independent of the specific details of a system.

To examine the domain evolution process under an electric field, domain structures at three different times from a phase-field simulation are shown in Fig. 4 (b–d). Compared with the initial domain structures in Fig. 4(a), the pink, red, and brown regions (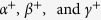
domains, which are unfavored by the applied electric field) in Fig. 4(b–c) shrink, and eventually become narrow 2-gons in Fig. 4(d). Movie-II (system size 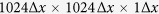) of the supplementary materials demonstrates the annihilation and creation of domain wall pairs, which are shown to be responsible for the appearance of isolated 4-gons^17^,^28^.

With the vortex cores fixed, the transition from type-I to type-II networks is caused by the annihilation and creation of domain wall pairs^28^. As shown in Fig. 2(e,f), when a domain wall pair is annihilated, a corresponding domain wall pair is created, resulting in the equal numbers of annihilated and created domain wall pairs. Interestingly, the gradient energy may increase due to the increase of the domain wall length, which gives rise to an energy barrier for the transition. For example, as shown in the inset of Fig. 4(c), the total length of the octopus-shaped domain walls (boundaries of the red domain) is longer than that of the initial domain walls indicated by dashed lines. The energy barrier may impede the annihilation of some domain wall pairs, giving rise to a coercive electric field. The value of the coercive field depends on the distance between the vortex and anti-vortex cores, i.e. the vortex density (for details, see supplementary information). The coercive field explains the existence of the 4-gons, 6-gons, and 8-gons of electric-field-unfavored domains in Fig. 3(f), even though the magnitude of the electric field in the simulation is 3 times larger than the experimental saturation field (The system size in the phase-field simulation is 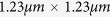, and the vortex density is higher than that of the experimental samples). The existence of the few 4-gons, 6-gons, and 8-gons will hardly change the above power-law distribution. It should be noted that the origin of the energy barrier is the topological deformation due to the domain wall movement, which is distinct from the conventional energy barrier during phase transitions.

Accompanied with the annihilation of a domain wall pair are two processes related to the N-gons: (1) an electric-field-favored *p*-gon and *q*-gon coalesce into a (*p*+*q*)-gon, and (2) an electric-field-unfavored *t*-gon splits into a *m*-gon and *k*-gon with 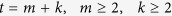, as shown in Fig. 2(e,f). The two processes keep repeating until equilibrium is reached. Note that the coalescence processes only happen among the same type of domains.

Since the vortex cores surrounding an electric-field-favored N-gon in type-I networks are subsets of those surrounding the corresponding N-gon in type-II networks, the occurrence of coalescence can be easily abstracted from the simulation results. Fig. 4(e) shows that the average coalescence occurrence is linearly dependent on N of N-gons. This indicates that the N-gons with a larger N have higher probability to grow, similar to a preferential attachment process. A preferential attachment process is a process that during the growth (adding nodes and corresponding connections to the network) of a network, the probability that an existing node builds connection with the new nodes is dependent on the degree of the existing node. It is shown that growth and preferential attachments are two fundamental mechanisms responsible for the scale-free feature in a complex network^30^. However, during the procedure described above, N-gons coalesce and split in the original graph, with the corresponding nodes merging and splitting in the dual graph, which are different operations from the network growth. Here we demonstrate that a coalescing and splitting process of N-gons with preferential attachments leads to a transition from lognormal to scale-free networks in the domain networks of hexagonal manganites.

The large variance of large N in Fig. 4(e) is due to the small numbers of corresponding N-gons, which indicates that N is not the only factor that affects the coalescence of a specific N-gon. Since an N-gon can only coalesce with the same type of N-gons (proper N-gons), the environment, i.e. the number of proper N-gons that are topologically close to it, determines the evolution of the N-gon. Other descriptors about the environment may be needed to determine the fate of a specific N-gon. However, statistically more proper N-gons are potentially close to an N-gon with a larger N, and the value of N is a critical factor for the coalescence process. Simplified simulations only maintaining the information of frequencies of N-gons show that a process with preferential attachments results in a power-law distribution, whereas that without preferential attachments maintains the lognormal distribution (see supplementary information).

Preferential attachment processes are similar to “proportionate growth”, and some natural processes following proportionate growth results in a lognormal distribution, so called “Gibrat’s law”^31^. In general, a process following Gibrat’s law gives rise to a lognormal or power-law distribution, depending on more specific details about the stochastic growth process^32^,^33^. In the situation of domain networks in hexagonal manganites, the lognormal distribution in type-I networks implies that preferential attachments may also exist in the coarsening process shown in Movie I.

In summary, we investigate the evolution of the statistical distribution of the N-gons in the domain networks of hexagonal manganites with electric fields using both phase-field simulations and experimental measurements. Lognormal- and power-law distributions are fitted for two types of domain networks, respectively. Preferential attachments (behaviors that the N-gons with a larger N have higher probability to coalesce with other N-gons) are shown to be responsible for the emergence of the power-law distribution. Our unique results provide new insights into understanding the kinetics and mechanism for the formation of different types of complex networks.

## Additional Information

**How to cite this article**: Xue, F. *et al.* Evolution of the statistical distribution in a topological defect network. *Sci. Rep.* **5**, 17057; doi: 10.1038/srep17057 (2015).

## Supplementary Material

Supplementary Information

Supplementary Information

Supplementary Information

## Figures and Tables

**Figure 1:**
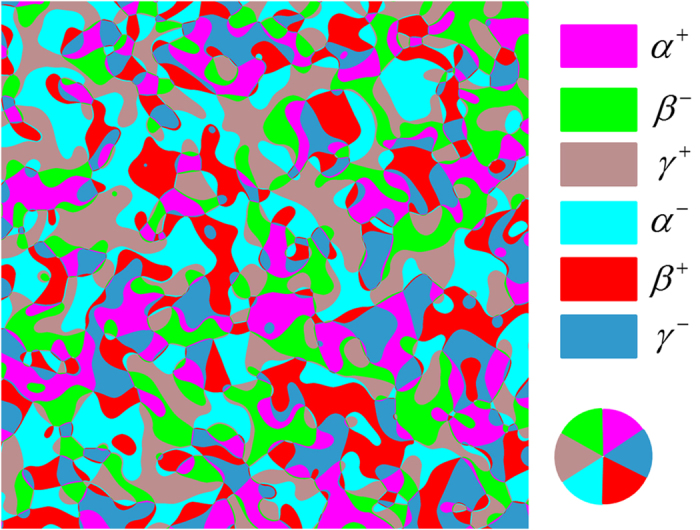
Domain patterns of a type-I network from a phase-field simulation. For better visualization, only 1/4 of the whole domain structure is shown. Different domains are denoted by different colors. The color wheel at lower right corner displays the arrangement of the six types of domains around a vortex.

**Figure 2:**
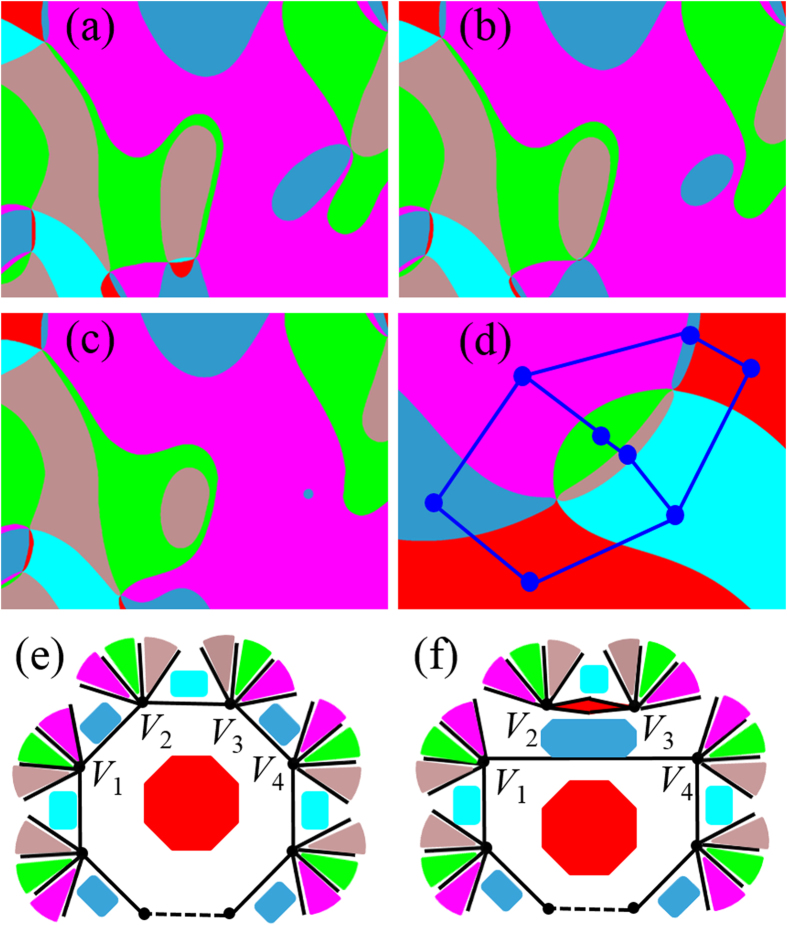
Topological condensation of vortex-antivortex pairs and domain wall pairs. (**a–c**) Annihilation of vortex-antivortex pairs from phase-field simulations, three zoomed snapshots of Movie-I. The brown and deep blue domains are two isolated gons resulting from the annihilation. **(d)** Schematics of a dual graph. The eight domains in the original graph correspond to eight nodes in the dual graph. The vortex-antivortex pair in the original graph corresponds to two 6-gons in the dual graph. **(e)** and **(f)** Schematics of annihilation and creation of domain wall pairs. The wall-pair *V*_1_*V*_2_ and *V*_3_*V*_4_ in **(e)** is replaced by the wall-pair *V*_1_*V*_4_ and *V*_2_*V*_3_ in **(f)**, accompanied by the splitting of a red N-gon and coalescing of two deep blue N-gons.

**Figure 3:**
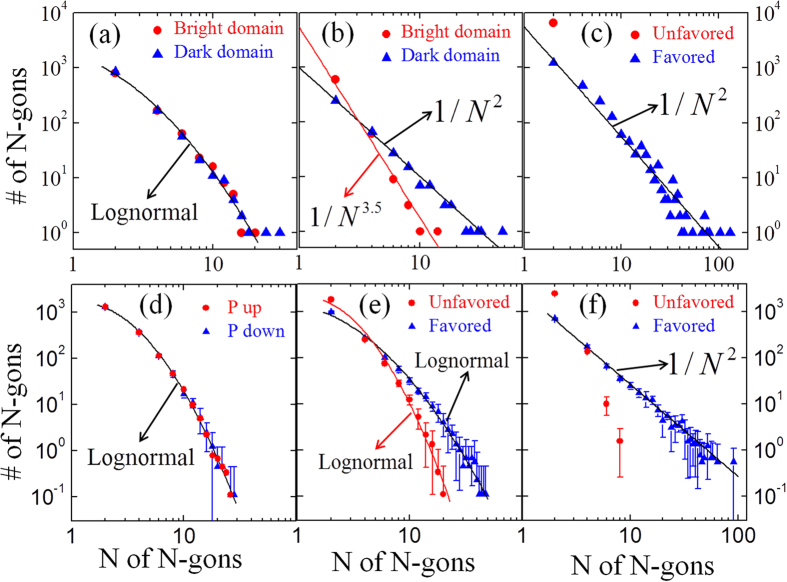
N-gon statistical analysis of experimental and phase-field simulation results. (**a**) A lognormal distribution within a type-I network in YbMnO_3_. (**b**) An intermediate network in an ErMnO_3_ crystal. (**c**) A power-law behavior within a type-II network in YMnO_3_. (**d**) A lognormal distribution of the type-I network without an electric field. (**e**) The N-gon distribution of the domain structures at an intermediate simulation time under an electric field. (**f**) A power-law distribution of the type-II network under an electric field. The data in (**d**–**f**) are average of nine parallel simulations with different random noises, and the error bars indicate the standard deviations.

**Figure 4:**
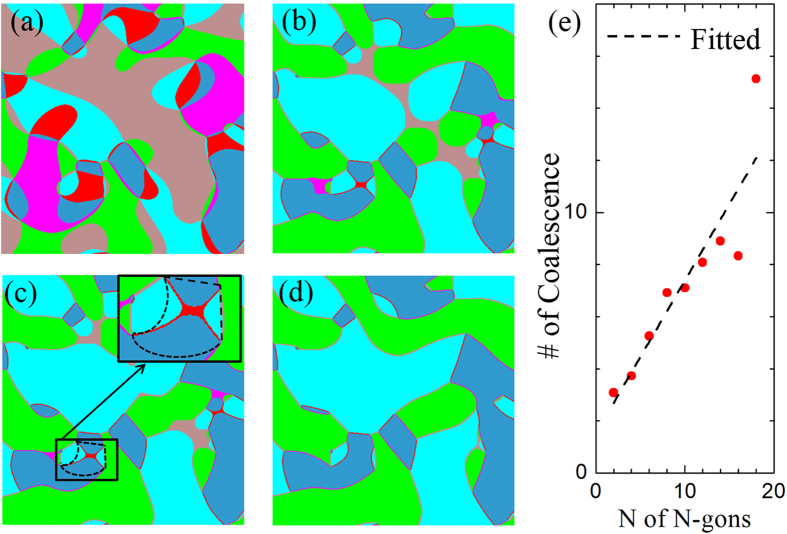
Transition from type-I to type-II networks under an electric field of 1200 *kV·cm*^−1^. (**a**) Initial domain patterns; (**b–d**) Domain patterns at three different simulation time steps. The inset of (**c**) shows an enlarged area, where the dashed line is the initial domain wall position in (**a**). (**e**) Average coalescence occurrence of the domains favored by the electric field as a function of N of N-gons. The red dots are averages of 5 parallel simulations starting different random noises, and the dashed line is drawn as a guide to the eye.
