# Supplementary material for: Evolution of the statistical distribution in a topological defect network
Source: Sci Rep. 2015 Nov 20;5:17057. doi: 10.1038/srep17057 (PMC4653636; doi:10.1038/srep17057)
Supplement: Supplementary Information [file srep17057-s1.doc]

**Evolution of the statistical distribution in a topological defect network**

Fei Xue,^1^ Xueyun Wang,^2^ Ion socolenco,^2^ Yijia Gu,^1^ Long-Qing Chen,^1^ and Sang-Wook Cheong^2,*^

^1^ Department of Materials Science and Engineering, The Pennsylvania State University, University Park, Pennsylvania 16802, USA

^2^ Rutgers Center for Emergent Materials and Department of Physics and Astronomy, Rutgers University, Piscataway, New Jersey 08854, USA

*Corresponding author: sangc@physics.rutgers.edu

**Supplementary Movies**

**Movie-I.gif | Vortex-antivortex annihilation from phase-field simulations.** The domain coarsening process starts from small random noise with the vortex cores mobile.

**Movie-II.gif | Domain structure evolution under an external electric field with the vortex cores fixed**. The topological changes are caused by domain wall annihilation and creation.

**Supplementary Information**

1. **Experimental N-gon analysis of three REMnO_3_ specimens**

All hexagonal REMnO_3_ crystal specimens were grown by a flux method: 10 mol% REMnO_3_ polycrystalline powders with 90 mol% Bi_2_O_3_ powders as flux were heated to 1250 °C and then cooled slowly to 800 °C in a platinum crucible. Plate-like crystals can be obtained with center meter size in the a-b plane and hundreds microns thickness along the c axis. Three specimens were used for N-gon analysis: YbMnO_3_ with type-I domains, ErMnO_3_ with an intermediate state between type-I and type-II domains, and YMnO_3_ with type-II domains. All specimens were chemically etched with phosphoric acid at 150 °C for 30 minutes to reveal the domain patterns on surfaces.

Large-range optical microscope and atomic force microscope (AFM) images were taken using a Zeiss Optical Microscope and a Nanoscope IIIA (Veeco), respectively. The full-range original optical microscope images can be found in the Supplementary Information of Ref. [1](#_ENREF_1). For better visualization, only parts of analyzed images are shown in Figs. S1-S3.


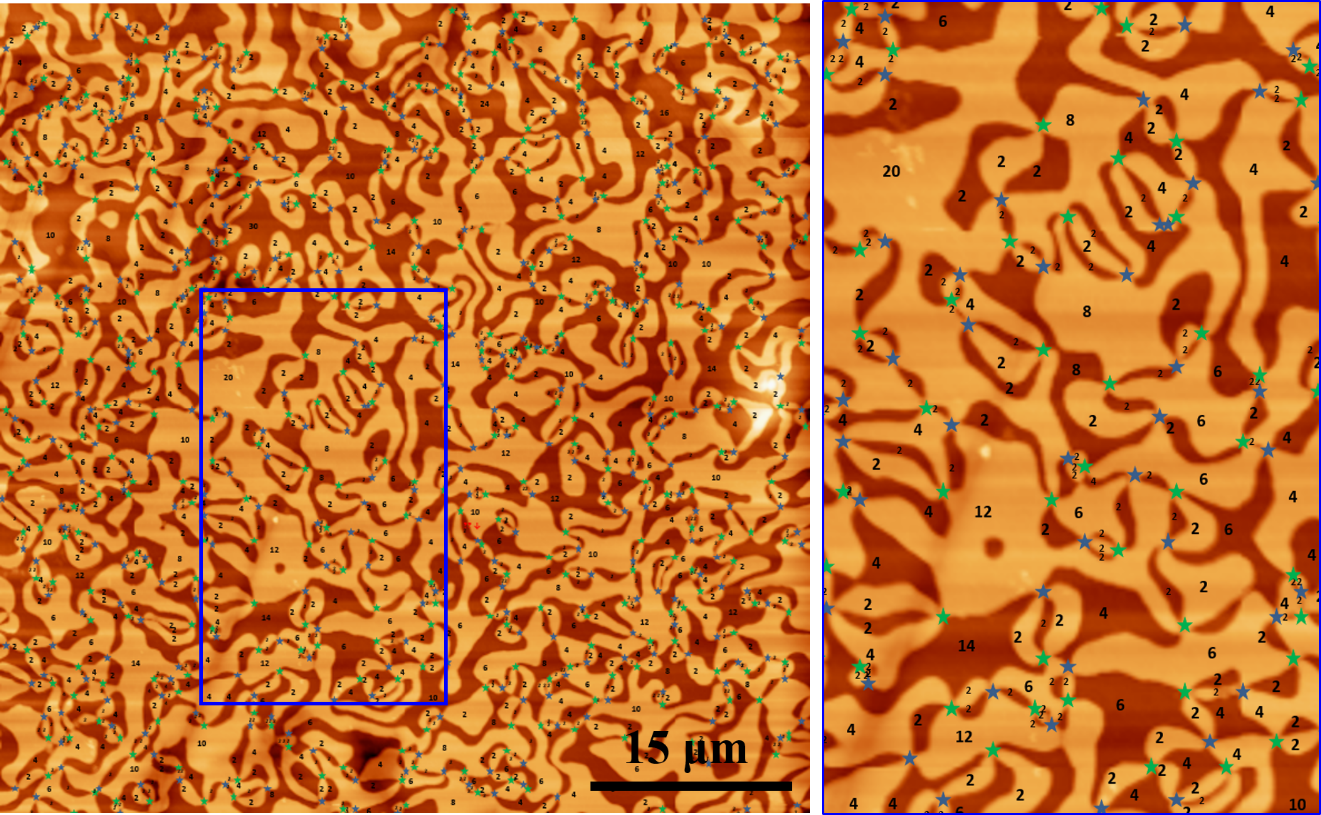


Figure S1 | Left panel: atomic force microscope scanning image on a chemically etched YbMnO_3_ crystal. Right panel: enlarged image of the region denoted by a blue rectangular. A green (blue) star denotes a vortex (antivortex), and an even integer N represents an N-gon.


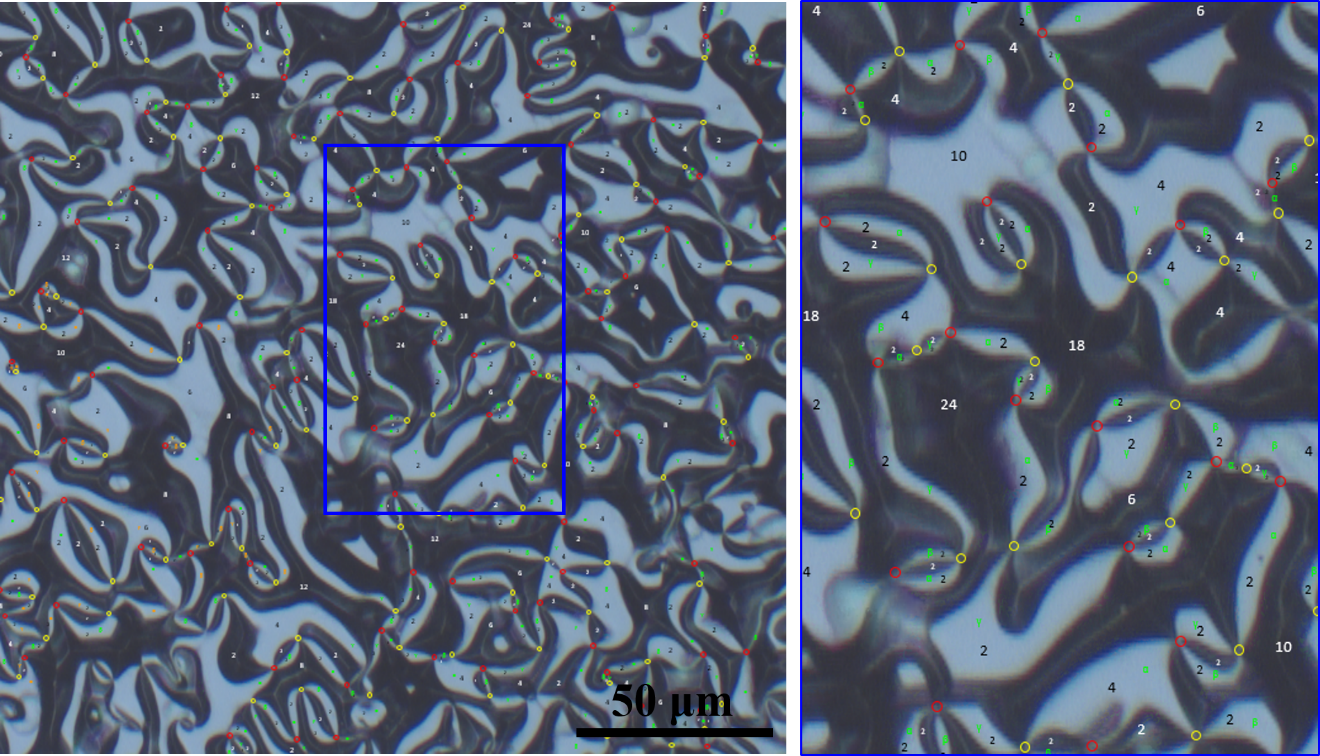


Figure S2 | Left panel: optical microscope image on a chemically etched ErMnO_3_ crystal. Right panel: enlarged image of the region denoted by a blue rectangular. A red (yellow) circle denotes a vortex (antivortex). An even integer N represents an N-gon. A Greek symbol$\alpha,\beta,\text{or }\gamma$ refers to the trimerization phase of the bright domain.


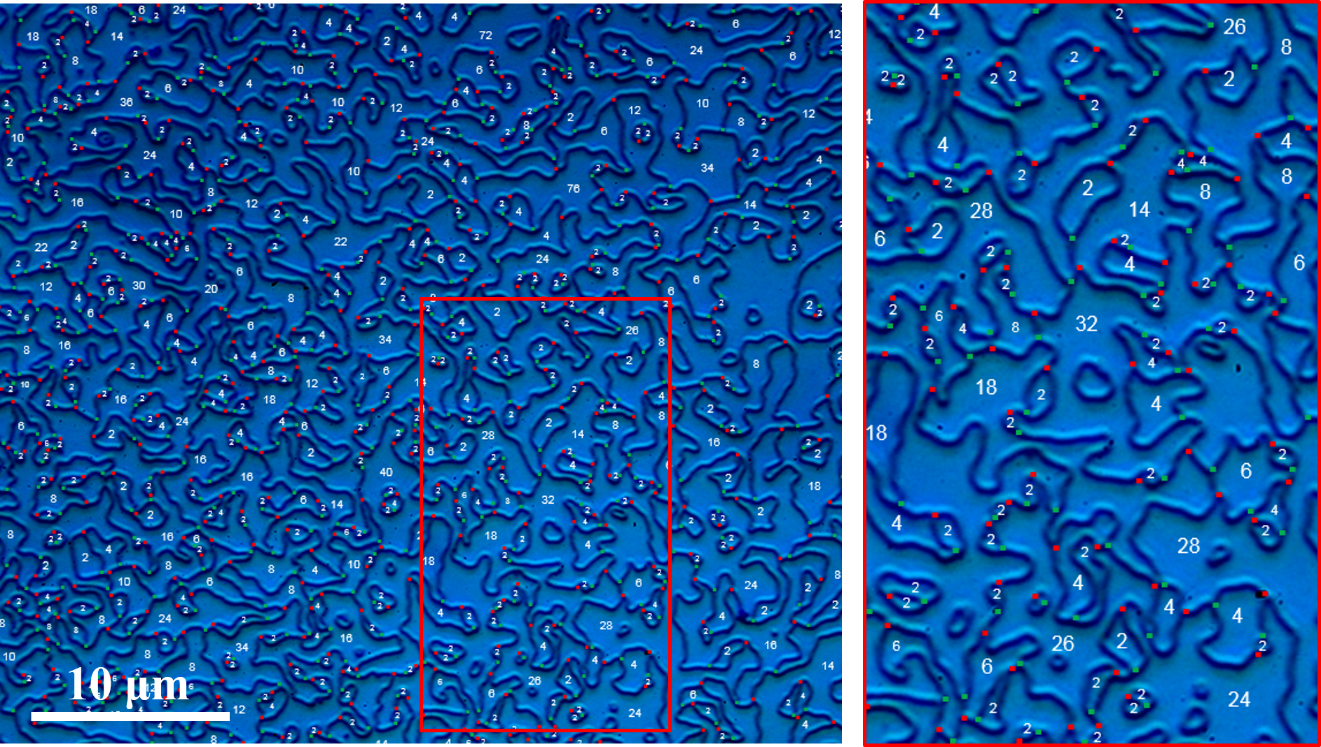


Figure S3 | Left panel: optical microscope image on a chemically etched YMnO_3_ crystal. Right panel: enlarged image of the region denoted by a red rectangular. A green (red) dot denotes a vortex (antivortex). An even integer N represents an electric-field-favored N-gon. The electric-field-unfavored N-gons are not labelled, since all the electric-field-unfavored domains are narrow 2-gons.

1. **Statistical analysis based on model selection techniques**

Firstly we give a brief introduction to the basic concept about model selection techniques. The details can be found in statistical text books such as Ref. [2](#_ENREF_2). In statistics, if a model M have $\upsilon$parameters $\theta=\{\theta_{j}\}$, likelihood is used to describe the probability of certain value $\theta_{j}$ given a sequence of outcome. For example, if we have statistical data$\{x_{1},x_{2},...,x_{n}\}$, the likelihood of a statistical model M is the probability that the model M produces the data $\{x_{1},x_{2},...,x_{n}\}$, and is given by

$L(M)=\prod_{i=1}^{n} P(x_{i}\left|M\right.)$ (S1)

where $P(x_{i}\left|M\right.)$is the probability distribution function. Taking logarithms on both sides yields the log-likelihood

$\text{lk}(M)=\sum_{i=1}^{n} \log(P(x_{i}\left|M\right.))$ (S2)

By maximizing equation (S2), we can estimate the value of $\theta_{j}$, which is so called “maximum likelihood estimates”. These values of $\theta_{j}$make the observed data more likely to occur than any other parameters.

Based on the maximum likelihood estimates, we can not only obtain the unknown parameters, but also determine how much better one model is at explaining the observed data than another model. Here we employ the Akaike information criterion (AIC), which is defined as

$\text{AIC}_{k}=2(-\text{lk}(M_{k})+\upsilon_{k})$ (S3)

where $\upsilon_{k}$is the number of parameters in model *k*. The model with the lowest AIC is chosen as the best model, which minimizes the information loss. The quantity $\exp[(\text{AIC}_{\min}-\text{AIC}_{k})/2]$is the relative likelihood of model *k*. If the difference of the AIC of two models is larger than 10, we can omit the model $\text{AIC}_{k}$with larger AIC, since exp[-5]=0.007, which means that the model *k* is 0.007 times as probable as the model with $\text{AIC}_{\min}$to minimize the information loss.

Following earlier statistical analysis on degree distributions of networks, we choose six models: exponential ($\exp[-\mu N]$), Gamma distribution ($N^{-\mu}\exp[-\sigma N]$), Poisson distribution ($\exp[-\mu]\mu^{N}/N!$), Stretched exponential ($N^{\mu-1}\exp[-\frac{N^{\mu}}{\sigma}]$), lognormal distribution ($\frac{\exp[-(\ln N-\mu)^{2}/2\sigma^{2}]}{\sigma N\sqrt{2\pi}}$), and power-law ($N^{-\xi}$). Table SI lists the log-likelihood values for the six models calculated using *mathematica* software. The simulation results are the average of nine simulations, and to obtain the maximum likelihood estimates, we round the average to the nearest integers.

Table SI. Maximum log-likelihoods for the six degree distributions as discussed in the text for different sets of data. The log-likelihoods with lowest AIC are indicated in bold. The maximum log-likelihoods for experiments and simulation type-II networks correspond to power law ($N^{-1.92}$) and ($N^{-2.22}$), respectively. The values in the brackets are log-likelihoods for power law ($N^{-2.00}$).

| Model | Exponential | Gamma | Poisson | Stretched Exponential | Lognormal | Power- Law |
| --- | --- | --- | --- | --- | --- | --- |
| Experiments type-I (Bright) (Bright) | -1513 | -1136 | -1487 | -1286 | **-969** | -983 |
| Experiments type-I (Dark) | -1552 | -1166 | -1548 | -1342 | **-966** | -978 |
| Experiments type-II | -4671 | -4571 | -5978 | -4658 | -4073 | **-3946** (-3952) |
| Simulations  type-I | -2600 | -1894 | -2505 | -2141 | **-1655** | -1771 |
| Simulations  type-II | -2014 | -1980 | -2687 | -2013 | -1687 | **-1499** (-1503) |

As shown in Table SI, lognormal distribution has the largest log-likelihood, i.e. lowest AIC, for type-I networks, whereas power-law distribution has the lowest AIC for type-II networks. The differences between the largest and second largest log-likelihood is larger than 10, and the differences between the lowest and second lowest AIC is larger than 20, which indicates that we can omit all the models except the one with the largest log-likelihoods. For the power-laws in type-II networks, the exponents are close to 2, and the log-likelihoods of the power law with exponents 2 are a little smaller than the maximum log-likelihoods, and much larger than those of other models. The power-laws with exponents 2 are shown in Figs. 3(c) and 3(f) of the main text, for the beauty and simplicity of physics.

Table SI also shows that the AICs of the lognormal and power-law distributions are lower than those of other four distributions for all the cases. Fig. S4 shows the fitting curves of the two models with the data of experimental and simulation results. The fittings based on maximum likelihood estimates look not so good in Figs. S4 (a) and S4 (c). This is because lognormal distributions are continuous distributions, and here N of N-gons is integers. Usually a discrete distribution can be well fitted by a continuous distribution when N is large, which is not the case here.


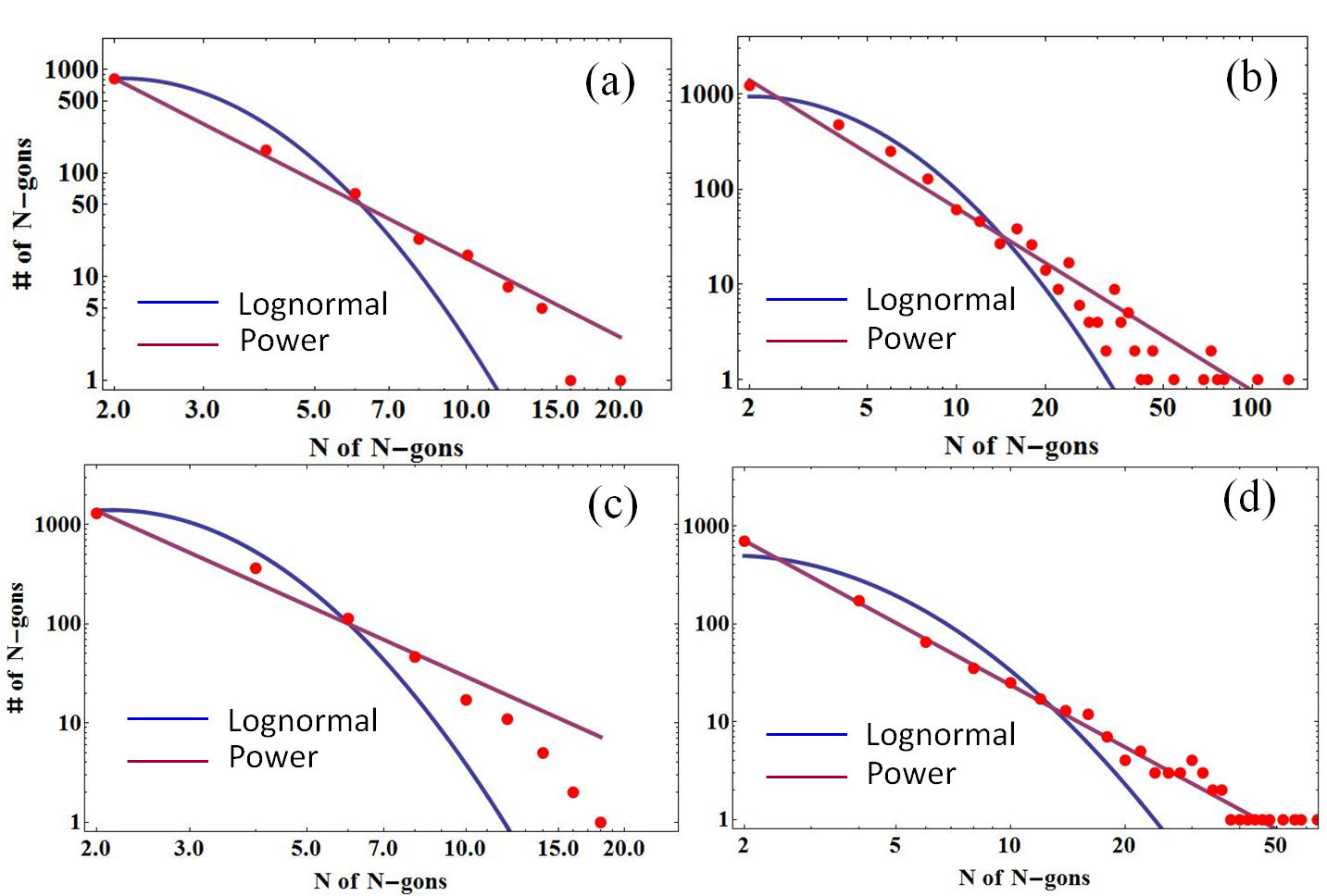


**Figure S4 |** Statistical distribution of N-gons based on maximum likelihood estimates. (a) Experimental type-I networks, (b) experimental type-II networks, (c) simulation type-I networks, and (d) simulation type-II networks. The fittings according to lognormal (blue), and power-law (purple) distributions are shown in each figure.

Usually a discrete distribution and a continuous one differ on the normalized constant, whereas their basic function forms are the same. Instead of developing new discrete lognormal distributions, we fit the curves based on the least-square methods in the log-log scale. Note that in the log-log scale, the power law is a straight line, whereas the lognormal distribution becomes a parabola. Thus lognormal fitting has an extra parameter to fit. As shown in Fig. S5, type-I networks are better fitted by the lognormal distributions, whereas type-II networks can be fitted by lognormal or power-law distributions. Note that the curves of the lognormal distributions look like a straight line, overlapping with those of the power-law distributions. We claim that the type-II networks follow the power-law distributions because the power-law fits better in the maximum likelihood estimates even though the least-square methods cannot distinguish.


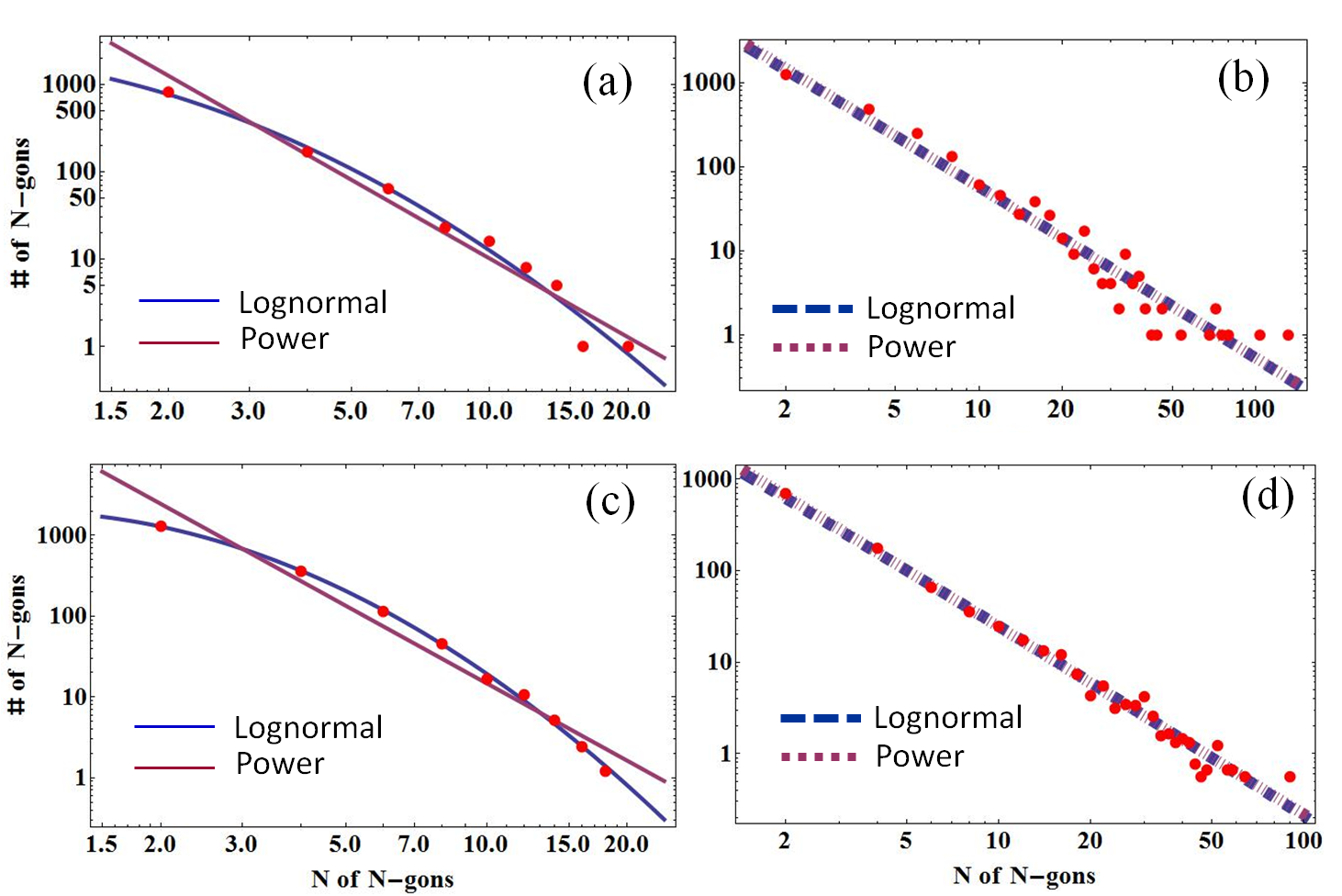


**Figure S5 |** Statistical distribution of N-gons based on least-square methods. (a) Experimental type-I networks, (b) experimental type-II networks, (c) simulation type-I networks, and (d) simulation type-II networks. The fittings according to lognormal (blue), and power-law (purple) distributions are shown in each figure. In (b) and (d), lognormal and power-law fittings overlap with each other, and dashed lines are used for better visualization. The exponents of the power-laws in (b) and (d) are 2.02, and 2.05, respectively.

In this work, we choose two out of six models based on the maximum likelihood estimates, and fit the parameters of the lognormal distributions based on the least-square methods. Comparing the lognormal and power-law distributions, both the maximum likelihood estimates and least-square methods show that type-I networks can be fitted by lognormal distributions, whereas type-II networks follow power-law distributions.

1. **Dual graph of large-range domain patterns**


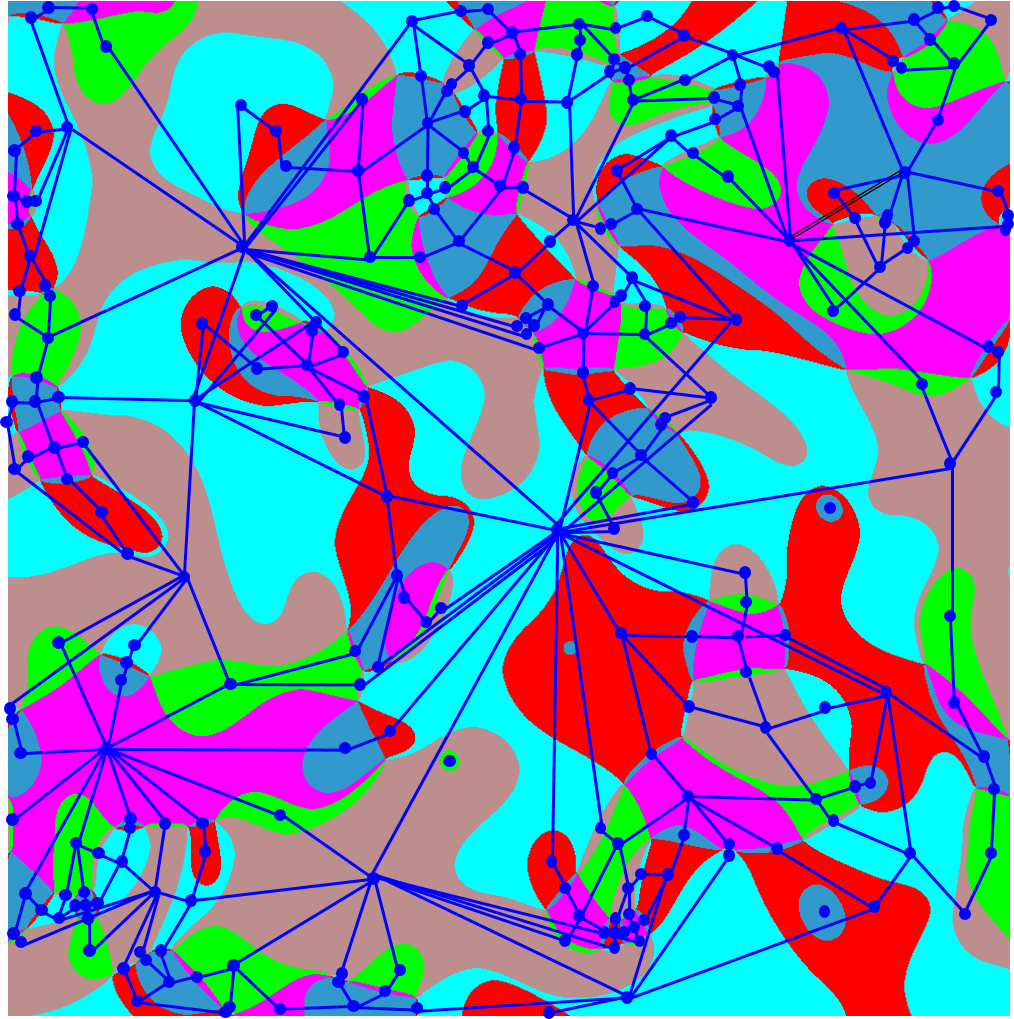


**Figure S6 |** Dual graph of large-range domain patterns from phase-field simulations (1/16 of the $\text{4096 } \Delta x\times\text{4096 } \Delta x\times1\Delta x$ system). In the dual graph, all the faces are 6-gons, and all the nodes have the degree of N (an even integer).

1. **Existence of coercive electric fields**

For simplicity, we use a 4-gon with a square shape to demonstrate the issue. As shown in Fig. S7, dots A, B, C and D are four vortex cores, which are fixed during the switching process. The solid lines represent the domain walls between $\alpha^{+}$and $\beta^{-}$domains, which are moved by an electric field favoring $\beta^{-}$domains. We assume the domain walls are arcs with the central angel $2\theta$and origins O and$\text{O}'$. The square ABCD has side length *l*. During the switching process, O and$\text{O}'$move from infinity to the midpoint of AB, and the central angel $2\theta$changes from 0 to $\pi/2$. Under an electric field along +*z* direction$E_{z}$, $\alpha^{+}$domain (pink color) shrinks and $\beta^{-}$domain (green color) expands. The total free energy of the square can be estimated by

$F=l^{2}f_{\alpha^{+}}+2l\frac{\theta}{\sin\theta}f_{wall}-(\frac{l^{2}\theta}{2\sin^{2}\theta}-\frac{l^{2}\cot\theta}{2})\cdot2E_{z}\cdot P_{z}$ (S4)

where $f_{\alpha^{+}}$is the energy density of single $\alpha^{+}$domain, $f_{wall}$is the domain wall energy density per length and $P_{z}$is the spontaneous polarization. Take the derivative of $F$with respect to $\theta$, we obtain

$\frac{\partial F}{\partial\theta}=2l\frac{1-\theta\cot\theta}{\sin\theta}(f_{wall}-\frac{E_{z}P_{z}l}{\sin\theta})$ (S5)

From $\frac{\partial F}{\partial\theta}=0$, we have $\sin\theta=\frac{E_{z}P_{z}l}{f_{wall}}$, which reaches its maximum value at $\theta=\frac{\pi}{2}$.

When $E_{z}\geq\frac{f_{wall}}{lP_{z}}$, $\frac{\partial F}{\partial\theta}$ is always less than 0, since $\frac{1-\theta\cot\theta}{\sin\theta}\geq0$. Therefore, the total free energy is monotonically decreasing and there are no energy barriers.

When $E_{z}<\frac{f_{wall}}{lP_{z}}$,$\frac{\partial F}{\partial\theta}=0$when $\theta=\arcsin\frac{E_{z}P_{z}l}{f_{wall}}$. At this situation, the annihilation of the domain-wall pair cannot be completed due to the energy barrier induced by the increase of domain wall energy.

Therefore, we obtain a coercive field $E_{z}=\frac{f_{wall}}{lP_{z}}$ , which is dependent on the magnitude of $l$, and the corresponding critical vortex density. The electric field with the magnitude smaller than the coercive field cannot change all the electric-field-unfavored domains into two 2-gons.


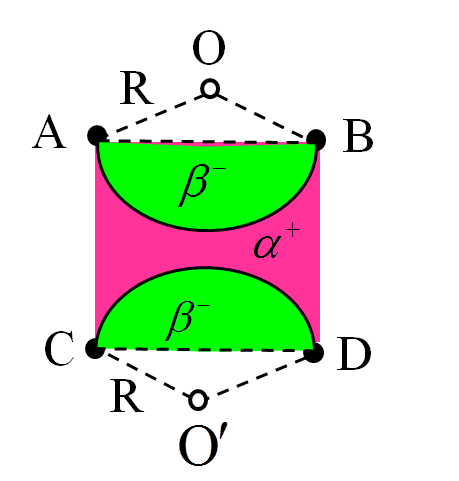


Figure S7 | Schematic plot of the switching process under an external electric field out of the plane of the page. Dots A, B, C, and D represent four vortex cores. Green domains are favored by the electric field and are growing. Pink domains are shrinking. Dots O and $O'$ are the virtual origins of the boundaries between the green and pink domains, i.e. arc AB and CD, and the radius is R.

1. **Simplified simulations of coalescing and splitting process**

To study the statistical behavior during the coalescing and splitting of the N-gons, we design a simplified simulation for the transition from type-I to type-II networks. It is assumed that the initial N-gons are free and separated, and the specific topological information about the connections of the N-gons is ignored. Among those separated N-gons, we randomly select one electric-field-unfavored N-gon and two electric-field-favored N-gons, and execute Process (1) and (2) as noted in the main text. Always selecting corresponding N-gons among the resulting N-gons from the last procedure, the above procedure is repeated for *S* times to split all the electric-field-unfavored N-gons into 2-gons, and *S* is given by $S=\frac{1}{2}\sum_{N=2}^{Max} (N-2)F_{N}$, where $F_{N}$ is the number of the initial electric-field-unfavored N-gons and Max is the largest number of N with a nonzero $F_{N}$. Then one simulation is completed, and the N-gons in the type-I network change into N-gons in a type-II network. With the initial N-gon statistical distribution of a type-I network, we perform the above simplified simulations for 100 times with different random selecting sequences, and take the average of the 100 simulations.


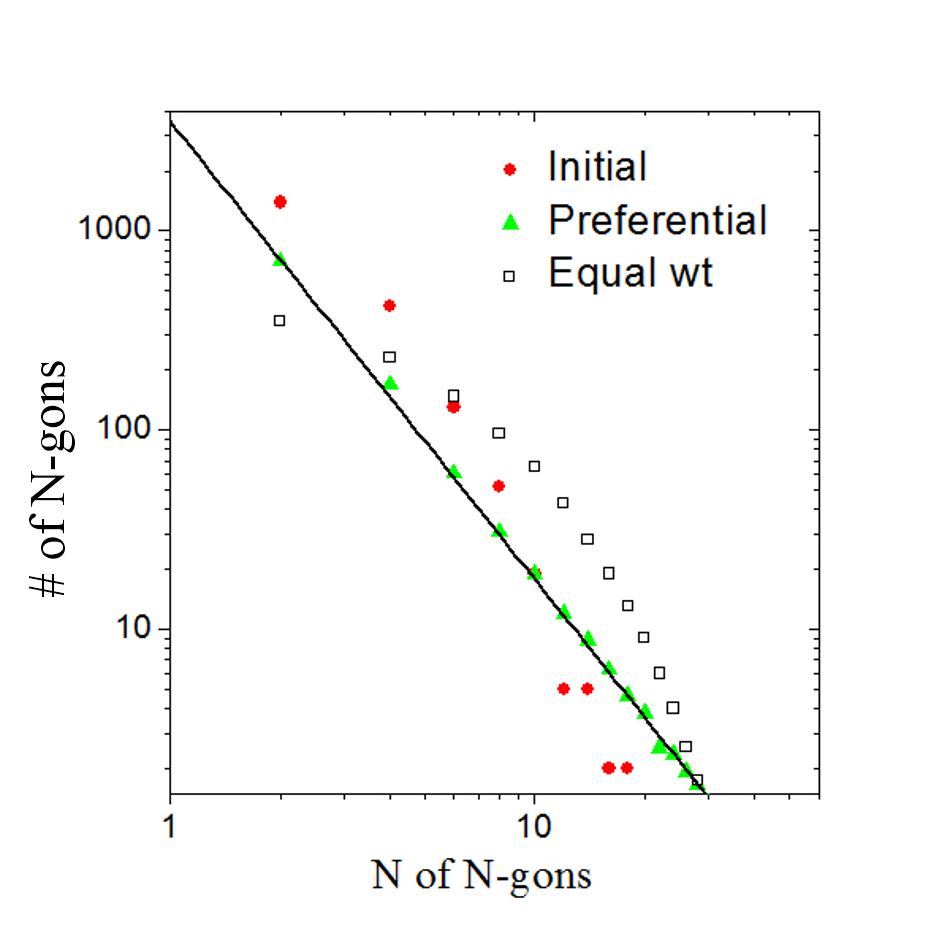


**Figure S8 |** Simplified simulations of the N-gon coalescing and splitting process. The N-gon distributions take into account only the domains favored by the electric field. The red filled circles correspond to the N-gon distribution of a type-I network. The open squares and filled triangles are the final N-gon distributions when the N-gons are selected with equal possibility, and with preferential attachments, respectively.

The resulting electric-field-favored N-gons show different statistical behaviors based on how the N-gons are selected. If all the N-gons are selected with equal possibility, it maintains as a lognormal distribution, plotted as open squares in Fig. S8 (we performed similar model selection process as in Section 2, and all the other models can be omitted). On the other hand, it shows the observed power-law distribution, as shown by the filled triangles in Fig. S8, if the N-gons are selected with the probability proportional to N.

1. **Area of a N-gon as a function of N**


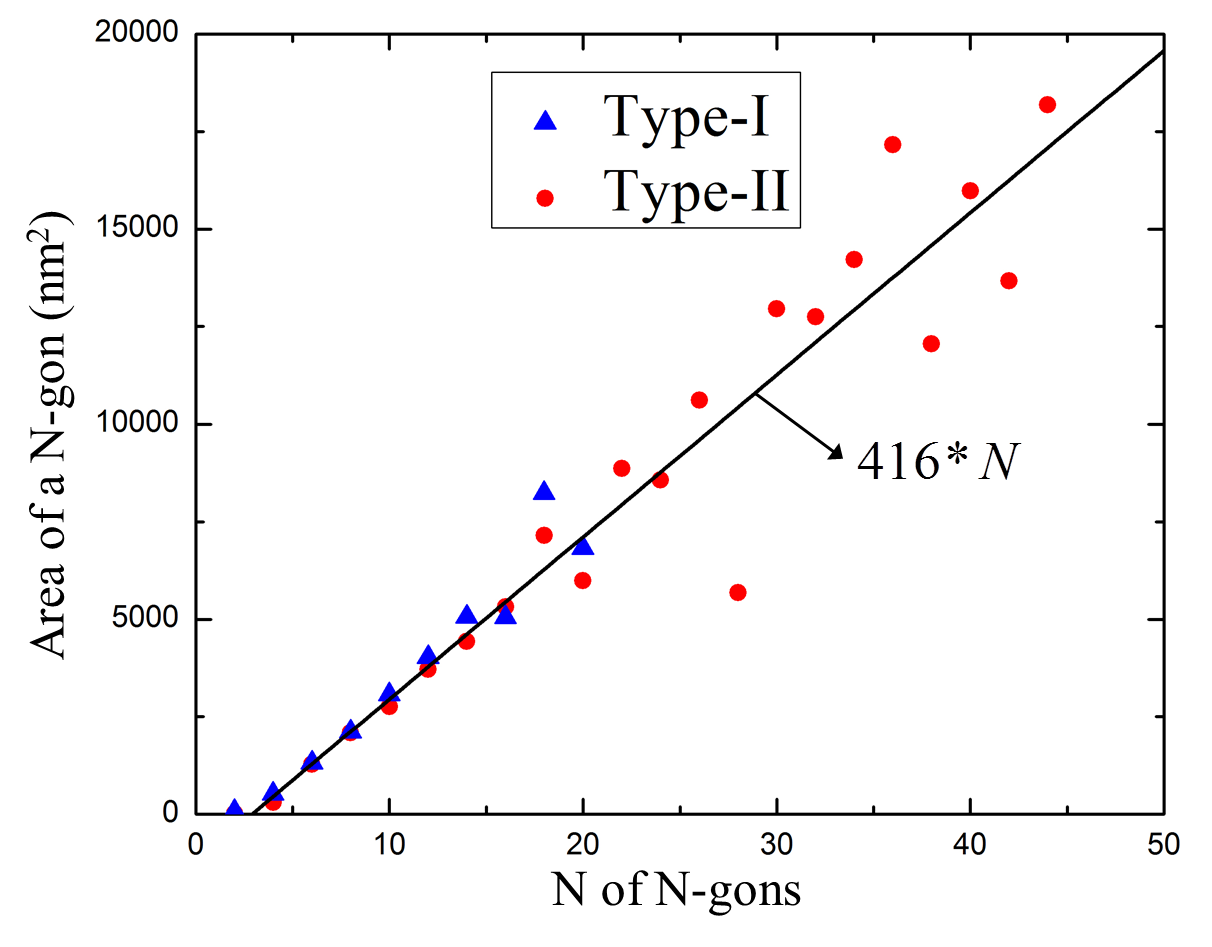


**Figure S9 |** Average area of an N-gon as function of N for the domain patterns from phase-field simulations. The linear function fits well with the data, which indicates that the area of an N-gon increases linearly with respect to N.

As shown in Fig. S9, the area of an N-gon has a linear relation with N. Therefore, whether the “area” or “N” of an N-gon is responsible for the preferential attachments does not make any differences. We believe that N has a direct relation with the preferential attachments, since N determines the number of neighbors. The more neighbors an N-gon has, the higher the probability that the N-gon coalesces with other N-gons is.

References:

1. Lin S-Z*, et al.* Topological defects as relics of emergent continuous symmetry and Higgs condensation of disorder in ferroelectrics. *Nature Physics* **10**, 970 (2014).

2. Davison A. *Statistical Models*. Cambridge University Press (2003).

3. Stumpf MP, Ingram PJ. Probability models for degree distributions of protein interaction networks. *EPL (Europhysics Letters)* **71**, 152 (2005).

4. Todor A, Dobra A, Kahveci T. Characterizing the topology of probabilistic biological networks. *IEEE/ACM Transactions on Computational Biology and Bioinformatics (TCBB)* **10**, 970-983 (2013).
