# Supplementary figures and images for: Evolution of the statistical distribution in a topological defect network
Source: Sci Rep. 2015 Nov 20;5:17057. doi: 10.1038/srep17057 (PMC4653636; doi:10.1038/srep17057)

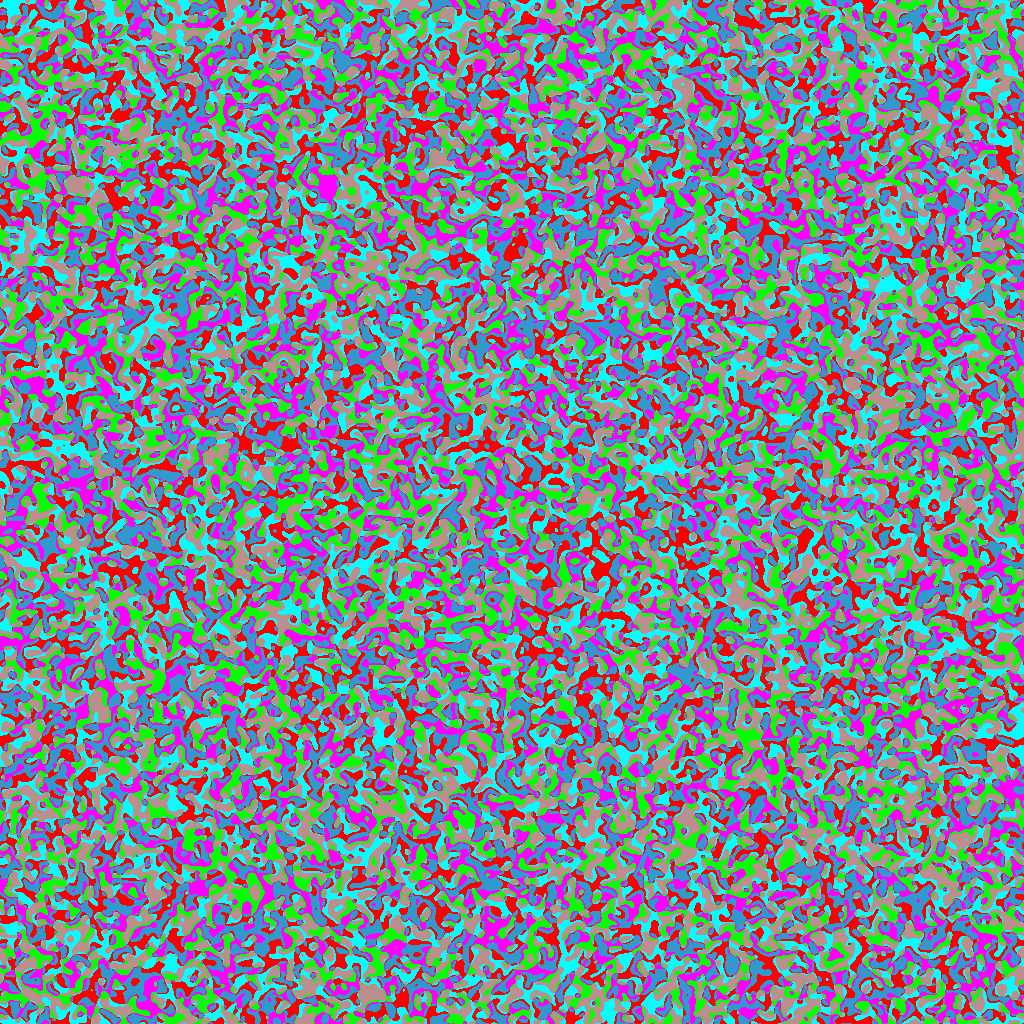

Supplement: Supplementary Information [file srep17057-s2.gif]

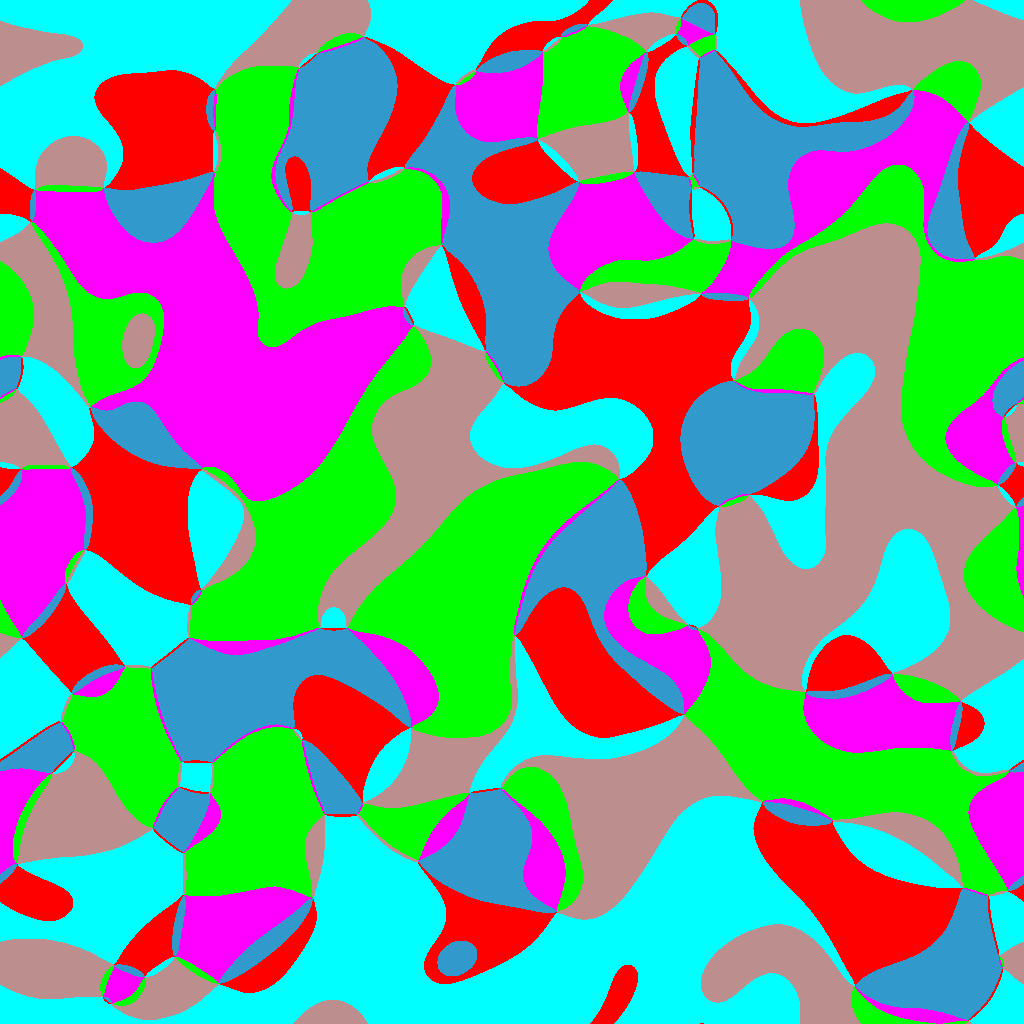

Supplement: Supplementary Information [file srep17057-s3.gif]
